# Correlation of Serum 1,5-AG with Uric Acid in Type 2 Diabetes Mellitus with Different Renal Functions

**DOI:** 10.1155/2019/4353075

**Published:** 2019-03-06

**Authors:** Kai Zhang, Bizhen Xue, Yuexing Yuan, Yao Wang

**Affiliations:** Department of Endocrinology, Zhongda Hospital, Institute of Diabetes, Medical School, Southeast University, No. 87 Dingjiaqiao Road, Nanjing 210009, Jiangsu Province, China

## Abstract

**Aim:**

Recent studies found that levels of serum uric acid (SUA) were positively associated with serum 1,5-anhydroglucitol (1,5-AG) in subjects with type 2 diabetes mellitus (T2DM). In the current study, we investigated the association between 1,5-AG and UA in T2DM patients with different renal functions.

**Methods:**

A total of 405 T2DM patients, 213 men and 192 women, participated in the study. Patients' clinical information was collected, and serum 1,5-AG, SUA, and other clinical characteristics were measured. Correlation analyses were carried out to analyze their correlation with serum 1,5-AG and SUA.

**Results:**

The male group showed higher levels of SUA than the female group (282.1 ± 91.2 and 244.7 ± 71.89 *μ*mol/L, respectively, *P* < 0.01). Pearson's correlation coefficients determine that SUA was positively associated with 1,5-AG in both men (*r* = 0.213, *P* < 0.05) and women (*r* = 0.223, *P* < 0.05), and such relationship can be influenced by the renal function. The positive association still existed with moderate impaired renal function. Moreover, 1,5-AG had a negative association with haemoglobin A1c (HbA1c) in T2DM subjects with eGFR ≥ 30 mL/min/1.73 m^2^ (*P* < 0.01).

**Conclusion:**

The positive association between SUA and 1,5-AG still exists in T2DM with moderate renal failure. 1,5-AG can still reflect the glucose levels in patients with CKD stages 1-3.

## 1. Introduction

1,5-Anhydroglucitol (1,5-AG), a 1-deoxy form of glucose analog, can reflect blood glucose levels over a period of 3-7 days as well as postprandial glucose [1, 2]. Dietary intake is the main source of 1,5-AG, and the levels of 1,5-AG are stable in healthy individuals [3]. During hyperglycemic periods, the blood glucose level is above the renal threshold and reabsorption of 1,5-AG is thought to be competed with glucose causing a decline in the serum 1,5-AG level [4, 5]. Besides, an earlier study reported that 1,5-AG is not likely to be affected by moderate renal impairment. Moreover, 1,5-AG is still a reliable blood glucose marker in type 2 diabetic patients with chronic kidney disease (CKD) stages 1-3 [6].

Serum uric acid (SUA), a weak organic acid, is the end product of purine metabolism in humans. SUA levels tend to rise with increasing blood glucose levels in the healthy and prediabetes population, while SUA levels decline in a patient with type 2 mellitus (T2DM) [7]. Similar to 1,5-AG, with long-term hyperglycemia, reabsorption of UA is also restricted causing a decrease in the SUA concentrations [8]. A previous study demonstrated that SUA was positively associated with 1,5-AG levels in patients with T2DM [9, 10]. In another research, it was reported that the previously mentioned positive association was stronger in T2DM patients than those without diabetes [11]. However, little information is available on the correlation between SUA and 1,5-AG in T2DM patients with different renal function.

In the current study, therefore, we investigated the correlation between SUA and 1,5-AG using classification by a CKD disease stage in T2DM subjects.

## 2. Research Design and Methods

### 2.1. Subjects

In this retrospective study, we enrolled 405 patients that were diagnosed with T2DM [according to guidelines of the World Health Organization (WHO) in1999] and treated from January 2013 to December 2015 in the Endocrinology and Metabolism Department of Southeast University-Affiliated Zhongda Hospital. Patients with cancer, liver dysfunction, or other illnesses affecting renal function such as renal artery stenosis were excluded, and patients using drugs like UA-lowering agents, diuretics, or fructose that might influence the level of UA were excluded. Type 1 diabetes and mitochondrial diabetes were excluded by clinical and immunological criteria.

### 2.2. Clinical and Biochemical Information

We collected patients' information: medication use, duration of diabetes, body mass index (BMI), blood pressure, and so on. Moreover, we sampled blood samples from all subjects between 7:00 and 9:00 a.m. and tested the serum concentrations of UA, creatinine, TC, TG, LDL, and HDL (Roche Diagnostic GmbH, Mannheim, Germany). Fasting blood glucose (FBG) and HbA1c were measured with an automated analyzer (Kyoto, Japan). 1,5-AG was estimated by using the GlycoMark assay (Tomen America, USA). Estimated glomerular filtration rate (eGFR) was calculated by the Modified Diet in Renal Disease (MDRD) study equation for the Chinese diabetic population as follows: eGFR = 1.211 × 170 sCr^−0.999^ × age^−0.176^ × BUN^−0.170^ × Alb^+0.318^× 0.762 (female) [12, 13]. Subjects with eGFR > 90 mL/min/1.73 m^2^ were categorized under stage 1 (normal kidney function), 60-89 mL/min/1.73 m^2^ under stage 2 (mild renal failure), 30-59 mL/min/1.73 m^2^ under stage 3 (moderate renal failure), and 15-29 mL/min/1.73 m^2^ under stage 4 (severely reduced kidney function). Nearly a quarter of the subjects in our study were found to have mild to moderate renal impairment including 62 patients with mild renal failure and 29 patients with moderate renal failure. Moreover, urine albumin-to-creatinine ratio (ACR) ≥ 30.0 mg/gCre is a risk factor for GFR decline and is included in the definition of CKD if it persists for ≥3 months [14, 15]. Therefore, ACR was also calculated for the evaluation of renal function and patients with ACR 0-29.9 mg/gCre were considered as normoalbuminuria, 30.0-299.9 mg/gCre as microalbuminuria, and ≥300 mg/gCre as macroalbuminuria.

### 2.3. Statistical Analysis

SPSS Software (version 21, SPSS Inc., Chicago, IL, USA) was utilized for the statistical analyses. Independent *t*-test, bivariate analysis, and multivariate regression were conducted to calculate the data. Continuous data are given as the mean ± SD, and categorical data are expressed as the percentage. *P* value < 0.05 was considered statistically significant.

## 3. Results

### 3.1. Subjects' Characteristics

The general clinical data gathered for each gender group are presented in Table 1. There were 192 women (including 71 premenopausal women and 121 postmenopausal women) and 213 men, and the female participants recruited in this study were older than male volunteers (62.9 ± 12.1 and 56.9 ± 13.8 years, respectively, *P* < 0.01). The male group showed higher levels of SUA than the female group (282.1 ± 91.2 and 244.7 ± 71.89 *μ*mol/L, respectively, *P* < 0.01), while eGFR, ACR, and serum 1,5-AG levels had no significant difference. Moreover, SUA levels among women with menopause were higher than those among premenopausal women (234.7 ± 66.57 and 247.9 ± 73.34 *μ*mol/L, respectively) (Table S1).

### 3.2. Correlations among the 1,5-AG Level and Other Clinical Variables

**Figure 1:**
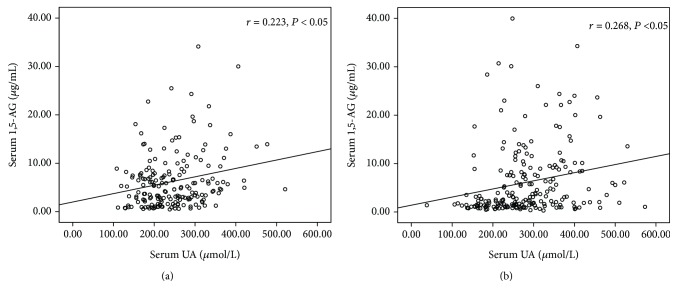
The relationship, as shown by Pearson's correlation coefficient, between levels of serum 1,5-AG and serum UA (a) in men (*r* = 0.213, *P* < 0.05) and (b) in women (*r* = 0.223, *P* < 0.05).

With an increase of 1,5-AG concentration, the glycemic levels like FPG and HbA1c significantly decreased and SUA levels were significantly elevated (Table 2). Therefore, we identified the correlations between serum 1,5-AG level and other clinical characteristics: age, HbA1c, and so on (Table 3). In both groups, 1,5-AG was significantly positively related with the duration of diabetes (*P* = 0.016 in female and *P* = 0.039 in male), UA (*P* = 0.038 in female and *P* = 0.03 in male), and HbA1c (*P* < 0.01 in both genders). In addition, as shown in Figure 1, Pearson's correlation coefficient revealed that 1,5-AG was positively associated with UA in each gender group (male: *r* = 0.213, *P* < 0.05; female: *r* = 0.223, *P* < 0.05), and the positive correlation also exists in premenopausal women (*r* = 0.256, *P* < 0.05) and postmenopausal women (*r* = 0.212, *P* < 0.05). Nevertheless, in both groups, there was no significant relationship between 1,5-AG and BMI or FPG.

### 3.3. Correlations between Serum 1,5-AG and SUA with Different Renal Functions

Next, we investigated the factors correlated with the positive association between serum 1,5-AG and SUA. The positive relationship still existed after adjusting possible influencing factors, such as age, BMI, blood pressure, lipids, HbA1c, and FBG, by multivariate regression analysis in both genders (Table 4). Interestingly, when taking eGFR and ACR into account, the previously mentioned positive association no longer existed (Table 4). Therefore, patients were divided into four or three groups using classification by eGFR or ACR in T2DM subjects. As indicated in Table 5 and Table S2, the association between SUA and 1,5-AG remains significant in diabetic patients with eGFR > 60 mL/min/1.73 m^2^ (*P* < 0.05) or with ACR < 300 mg/gCre (*P* < 0.01).

### 3.4. Correlation between 1,5-AG and HbA1c in Different Renal Functions

Finally, regression analyses were conducted to investigate the correlation between 1,5-AG and HbA1c in T2DM with different renal functions. As shown in Table 5, 1,5-AG had a negative association with HbA1c in T2DM subjects with eGFR ≥ 30 mL/min/1.73 m^2^ (*P* < 0.01).

## 4. Discussion

The current study provides the evidence that SUA in relation to 1,5-AG in T2DM subjects with CKD stages 1-2. The relation was noted in both men and women. Furthermore, the negative association between HbA1c and 1,5-AG still exists in T2DM patients with eGFR ≥ 30 mL/min/1.73 m^2^, and 1,5-AG remains a reliable glycemic control marker.

UA is a product of the metabolic breakdown of purine nucleotides and is excreted and absorbed via the kidney. When the concentration of blood glucose reaches a kidney's reabsorptive threshold, glucose will be excreted in the urine causing the decline of reabsorption of UA, and any impairment of kidney function can result in hyperuricemia [16–18]. In the present study, SUA levels in the female group are lower compared to those in the male group, which is consistent with previous observations [19–21]. Moreover, in our research, SUA levels of menopause women were also higher than those of premenopausal women. One possible explanation for these results is that estrogens may promote renal clearance of UA. And actually, studies have demonstrated that administration of estrogen therapy to males can decrease SUA levels [22–24]. In addition, the positive relationship between SUA and 1,5-AG in the male and female groups has been reported in previous studies; however, little is known about the association in the premenopausal and postmenopausal women. Since hormonal situation may have an influence on the relationship between serum 1,5-AG and UA, we analyzed the relationship of female participants based on hormonal situation. Interestingly, as indicated in Figure S1, the positive correlation still existed in both premenopausal and postmenopausal women. Besides, similar to SUA, 1.5-AG levels of menopause women were also higher than those of premenopausal women. One possible explanation to illustrate the result is that estrogens may also promote renal clearance of 1,5-AG.

Although the positive relation between SUA and 1,5-AG has been found in a large number of studies, little is known about the association in patients with chronic renal disorder. In our present study, multivariate analyses found a positive association between SUA and 1,5-AG, independent of HbA1c and FBG. Interestingly, when taking eGFR and ACR into account, the significant association mentioned previously disappeared. Therefore, the subjects were classified into different groups based on eGFR or ACR. We found that 1,5-AG and SUA still associated positively in T2DM patients with eGFR ≥ 60 mL/min/1.73 m^2^ or with ACR < 300 mg/gCre. However, this positive association no longer existed when eGFR declined under 60 mL/min or ACR ≥ 300 mg/gCre. Consequently, we suggest that the renal function can affect the association between SUA and 1,5-AG and some explanations may illustrate the results. Firstly, both 1,5-AG and UA were competitively reabsorbed with renal glucose in the proximal tubules [25, 26], and it has been reported that renal disorder can affect UA transport in diabetic patients and 1,5-AG was negatively associated with kidney function [27, 28]. Secondly, a previous study reported that a solute carrier (SLC) 2A9, also a fructose transporter, was identified to be a UA transporter [29]. Moreover, dapagliflozin, a sodium-glucose cotransporter (SGLT2) inhibitor, significantly decreased SUA compared with placebo and might also decrease serum 1,5-AG, indicating that 1,5-AG and UA may share the same renal transporter, and we suppose that impairment of the transporter in patients with moderate renal disorder may result in the disappearance of the positive association between 1,5-AG and SUA [30, 31]. Furthermore, 1,5-AG is positively correlated with SUA in T2DM patients and may share a common renal transporter with UA; therefore, 1,5-AG cannot reflect blood glucose levels accurately in patients who take uricosuric medicine like benzbromarone.

Currently, HbA1C is the “gold standard” for identifying the overall blood glucose control; however, 1,5-AG which can reflect rapid changes in glycemia is more accurate than HbA1C [32]. Therefore, 1,5-AG as a blood glucose marker can provide complementary information to HbA1C. Moreover, in our previous observation, we demonstrated that serum 1,5-AG is a sensitive and specific glycemic control marker and can identify diabetes in populations [33]. However, unlike HbA1c, multiple factors such as diet and renal function may limit the comparison of 1,5-AG levels between individuals. Some researchers reported that 1,5-AG cannot be used to predicate blood glucose in patients that suffer from impaired renal function [34]. In the present study, the subjects were classified into different groups by eGFR, and we found that 1,5-AG was still negatively associated with HbA1c in T2DM subjects with eGFR ≥ 30 mL/min/1.73 m^2^. Considering these results, we conclude that 1,5-AG remain a reliable glycemic control marker even if patients suffer mild or moderate renal dysfunction, which is consistent with a previous observation [35]. In addition, in the current study, we showed that serum 1,5-AG levels were not influenced by eGFR, whereas a previous study reported that 1,5-AG was negatively correlated with kidney function, which might be owing to an age-associated decrease in the renal glucose threshold [28].

The limitations of the present study include the following: our study is a cross-sectional design in a single center with a high risk of selection bias; the consumption of meat or alcohol was not examined, and urate oxidase was not assessed.

Collectively, the present study provides the evidence that 1,5-AG was positively associated with SUA in T2DM patients with mild and moderate renal failure. In addition, 1,5-AG can reflect the glucose metabolic situation in patients with CKD stages 1-3.

## Figures and Tables

**Table 1:** Clinical characteristics of the study participants.

Characteristics	Female (*n* = 192)	Male (*n* = 213)	*P* value
Age (years)	62.9 ± 12.1	56.9 ± 13.8	<0.001
Diabetes duration (years)	8.7 ± 6.8	6.5 ± 6.1	<0.001
BMI (kg/m^2^)	24.4 ± 2.9	24.6 ± 3.4	0.466
SBP (mmHg)	133.5 ± 17.1	130.8 ± 16	0.106
DBP (mmHg)	78 ± 8.4	79.6 ± 10.2	0.083
Serum creatinine (mg/dL)	0.66 ± 0.19	0.82 ± 0.2	<0.001
Blood urea nitrogen (mg/dL)	15.4 ± 4.78	16.29 ± 4.8	0.052
Serum albumin (g/dL)	4.4 ± 1.32	4.5 ± 1.94	0.548
ACR (mg/gCre)	54.3 ± 69.46	52.56 ± 72.38	0.805
eGFR (mL/min/1.73 m^2^)	114.6 ± 31.2	115.1 ± 31.8	0.873
UA (*μ*mol/L)	244.7 ± 71.89	282.1 ± 91.2	<0.001
FBG (mmol/L)	9.1 ± 3.5	9.2 ± 4.2	0.708
1,5-AG (*μ*g/mL)	6.2 ± 6.01	6.1 ± 7.2	0.881
HbA1C (%)	9.18 ± 2.33	9.34 ± 2.56	0.517
HDL (mmol/L)	1.19 ± 0.52	1.1 ± 0.51	0.081
LDL (mmol/L)	2.72 ± 0.99	2.54 ± 0.96	0.059
TC (mmol/L)	4.83 ± 1.1	4.61 ± 1.05	0.386
TG (mmol/L)	1.99 ± 1.3	1.97 ± 1.5	0.886

SBP: systolic blood pressure; DBP: diastolic blood pressure; HDL: high-density lipoprotein; LDL: low-density lipoprotein; TC: total cholesterol; TG: triglycerides.

**Table 2:** Comparison of variables according to quartiles of serum 1,5-AG levels.

Quartiles of 1,5-AG (*μ*g/mL)	<1.45	1.45–3.35	3.35–8.70	>8.70	*P* value
Age (years)	56.1 ± 14.3	57.6 ± 13.6	61.6 ± 11.8	63.6 ± 12.3	<0.001
Diabetes duration (years)	6.1 ± 5.6	8.1 ± 6.8	9.2 ± 7.4	6.8 ± 5.7	0.003
BMI (kg/m^2^)	24.84 ± 3.43	24.05 ± 3.28	24.29 ± 2.61	24.93 ± 3.38	0.149
SBP (mmHg)	129 ± 13.4	130.6 ± 16	134.5 ± 17.3	134.3 ± 18.5	0.041
DBP (mmHg)	78.2 ± 8.9	78.8 ± 10.4	80.1 ± 9.8	78.2 ± 8.6	0.422
Serum creatinine (mg/dL)	0.7 ± 0.16	0.73 ± 0.21	0.76 ± 0.26	0.79 ± 0.2	0.021
Blood urea nitrogen (mg/dL)	16 ± 4.49	16.29 ± 5.06	15.45 ± 4.78	15.5 ± 4.49	0.464
Serum albumin (g/dL)	4.3 ± 1.37	4.5 ± 1.43	4.6 ± 1.52	4.3 ± 1.34	0.547
ACR (mg/gCre)	57.06 ± 70.53	54.10 ± 69.27	54.60 ± 71.52	53.10 ± 68.92	0.786
eGFR (mL/min/1.73 m^2^)	117.48 ± 27.17	114.32 ± 31.78	116.12 ± 35.97	110.37 ± 35.15	0.442
UA (*μ*mol/L)	233.95 ± 84.88	247.91 ± 82.58	277.77 ± 83.59	296.56 ± 82.96	<0.001
FBG (mmol/L)	12.13 ± 4.27	9.43 ± 3.16	8.13 ± 3.67	6.86 ± 1.84	<0.001
1,5-AG (*μ*g/mL)	0.96 ± 0.28	2.32 ± 0.58	5.92 ± 1.53	16.63 ± 6.89	<0.001
HbA1C (%)	11.97 ± 1.99	9.97 ± 1.8	8.09 ± 1.14	7.00 ± 1.11	<0.001
HDL (mmol/L)	1.19 ± 0.39	1.17 ± 0.31	1.12 ± 0.28	1.09 ± 0.29	0.157
LDL (mmol/L)	2.69 ± 0.71	2.78 ± 1.21	2.52 ± 0.79	2.51 ± 1.10	0.140
TC (mmol/L)	4.92 ± 0.98	4.83 ± 1.07	4.65 ± 1.25	4.45 ± 0.92	0.180
TG (mmol/L)	2.07 ± 1.64	1.92 ± 1.45	1.81 ± 1.09	1.92 ± 1.31	0.616

SBP: systolic blood pressure; DBP: diastolic blood pressure; ACR: urine albumin-to-creatinine ratio; HDL: high-density lipoprotein; LDL: low-density lipoprotein; TC: total cholesterol; TG: triglycerides.

**Table 3:** Stepwise multiple linear regression analysis with serum 1,5-AG levels as the dependent variable and clinical characteristics as independent variables.

Variables	Female		Male	
	*β*	*P* value	*β*	*P* value
Age (years)	0.074	0.271	0.025	0.722
Diabetes duration (years)	−0.163	0.016	−0.134	0.039
BMI (kg/m^2^)	−0.050	0.409	−0.032	0.600
SBP (mmHg)	0.118	0.115	0.042	0.618
DBP (mmHg)	−0.119	0.088	−0.022	0.792
Serum creatinine (mg/dL)	−0.071	0.432	−0.057	0.436
Blood urea nitrogen (mg/dL)	0.003	0.962	−0.109	0.091
ACR (mg/gCre)	0.040	0.570	0.014	0.816
eGFR (mL/min/1.73 m^2^)	−0.097	0.142	−0.084	0.178
HbA1_c_ (%)	−0.528	<0.001	−0.563	<0.001
FBG (mmol/L)	−0.122	0.074	−0.094	0.140
UA (*μ*mol/L)	0.143	0.038	0.148	0.030
HDL (mmol/L)	−0.081	0.182	0.044	0.507
LDL (mmol/L)	0.075	0.282	−0.004	0.952
TC (mmol/L)	−0.071	0.333	−0.048	0.522
TG (mmol/L)	0.019	0.870	−0.080	0.246

SBP: systolic blood pressure; DBP: diastolic blood pressure; ACR: urine albumin-to-creatinine ratio; HDL: high-density lipoprotein; LDL: low-density lipoprotein; TC: total cholesterol; TG: triglycerides.

**Table 4:** Multivariate regression analyses of factors correlated with the positive association between serum 1,5-AG and serum UA.

	Female		Male	
	*r*	*P* value	*r*	*P* value
Model 1	0.294	<0.001	0.223	<0.001
Model 2	0.276	<0.001	0.224	<0.001
Model 3	0.205	0.007	0.216	<0.001
Model 4	0.146	0.057	0.110	0.130

^1^Model 1: adjusted for age and diabetes duration; model 2: model 1 plus DBP, SBP, BMI, HDL, LDL, TC, and TG; model 3: model 2 plus HbA1c and FBG; model 4: model 3 plus eGFR and ACR. ^2^SBP: systolic blood pressure; DBP: diastolic blood pressure; ACR: urine albumin-to-creatinine ratio; HDL: high-density lipoprotein; LDL: low-density lipoprotein; TC: total cholesterol; TG: triglycerides.

**Table 5:** Regression analyses of correlations between kidney function and 1,5-anhydroglucitol, HbA1c, and uric acid.

eGFR (mL/min/1.73 m^2^)	No. of subjects	Slope	1,5-AG vs. HbA1c		Slope	1,5-AG vs. UA	
			Intercept	*P* value		Intercept	*P* value
>120	188	−0.663	24.024	<0.001	0.242	1.117	0.001
90-119.99	126	−0.583	20.758	<0.001	0.175	2.102	0.050
60-89.99	62	−0.618	28.691	<0.001	0.279	1.339	0.028
30-59.99	29	−0.639	22.869	<0.001	0.143	4.004	0.458

## Data Availability

All the data supporting the results are shown in the paper and can be applicable from the corresponding author.

## Supplementary Materials

Supplementary Materials Supplementary Material Description: clinical characteristics of female participants based on menopausal status are shown in Table S1, and SUA levels among women with menopause were higher than those among premenopausal women. Table S2 shows the correlations between 1,5-anhydroglucitol and uric acid with different ACR. The association between SUA and 1,5-AG remains significant in diabetic patients with ACR < 300 mg/gCre (*P* < 0.01). The relationship between levels of serum 1,5-AG and serum UA in premenopausal women (*r* = 0.256,*P* < 0.05) and in postmenopausal women (*r* = 0.212,*P* < 0.05) are shown by Pearson's correlation coefficient in Figure S1.
