# Supplementary material for: Correlation of Serum 1,5-AG with Uric Acid in Type 2 Diabetes Mellitus with Different Renal Functions
Source: Int J Endocrinol. 2019 Mar 6;2019:4353075. doi: 10.1155/2019/4353075 (PMC6431393; doi:10.1155/2019/4353075)

**Supplementary Material**

**Table S1** Clinical characteristics of female participants **based on menopausal status**

| Characteristics | Premenopausal  women (n=71) |  | Postmenopausal women (n=121) |  | P-value |
| --- | --- | --- | --- | --- | --- |
| Age(years) | 48.1±6.93 |  | 68.2±8.66 |  | ＜0.001 |
| Diabetes duration(years) | 6.2±6.11 |  | 9.5±6.73 |  | ＜0.001 |
| BMI(kg/m^2^) | 24.4±2.9 |  | 24.3±2.78 |  | 0.452 |
| SBP(mmHg) | 125.3±11.21 |  | 136.4±17.8 |  | ＜0.001 |
| DBP(mmHg) | 76.2±7.65 |  | 78.6±8.51 |  | 0.085 |
| Serum creatinine(mg/dL) | 0.72±0.29 |  | 0.77±0.18 |  | 0.141 |
| Blood urea nitrogen (mg/dL) | 14.33±4.52 |  | 15.73±4.66 |  | 0.106 |
| Serum albumin (g/dL) | 4.3±1.26 |  | 4.5±1.34 |  | 0.354 |
| ACR(mg/gCre) | 42.7±51.12 |  | 58.2±74.18 |  | 0.189 |
| eGFR(ml/min/1.73 m^2^) | 121.8±29.65 |  | 112.1±31.36 |  | 0.068 |
| UA(μmol/L) | 234.7±66.57 |  | 247.9±73.34 |  | 0.218 |
| FBG(mmol/L) | 9.2±3.23 |  | 9±3.59 |  | 0.793 |
| 1,5-AG(μg/mL) | 5.6±5.70 |  | 6.4±6.21 |  | 0.306 |
| HbA1C(%) | 9.4±2.41 |  | 9.1±2.30 |  | 0.537 |
| HDL(mmol/L) | 1.2±0.37 |  | 1.2±0.30 |  | 0.742 |
| LDL(mmol/L) | 2.7±0.76 |  | 2.7±1.06 |  | 0.640 |
| TC(mmol/L) | 4.8±1.0 |  | 4.8±1.12 |  | 0.768 |
| TG(mmol/L) | 2.2±1.62 |  | 1.9±1.06 |  | 0.190 |
| SBP: Systolic blood pressure; DBP: Diastolic blood pressure; HDL: high density lipoprotein; LDL:  low density lipoprotein; TC: total cholesterol; TG: triglycerides | | | | | |

**Table S2 Correlations between 1,5-anhydroglucitol and uric acid with different ACR**

|  | | 1,5-AG vs UA | | |
| --- | --- | --- | --- | --- |
| ACR(mg/gCre) | No.subjects | r |  | P-value |
| ＜30 | 222 | 0.272 |  | <0.01 |
| 30-299 | 169 | 0.274 |  | <0.01 |
| ≥300 | 10 | 0.122 |  | 0.878 |

ACR: Urine albumin-to-creatinine ratio;

**Fig .S1**  The relationship, as shown by the person’s correlation coefficient, between levels of serum 1,5-AG and serum UA (a) in premenopausal women (r=0.256, *P*<0.05) (b) in postmenopausal women (r=0.212, *P*<0.05)


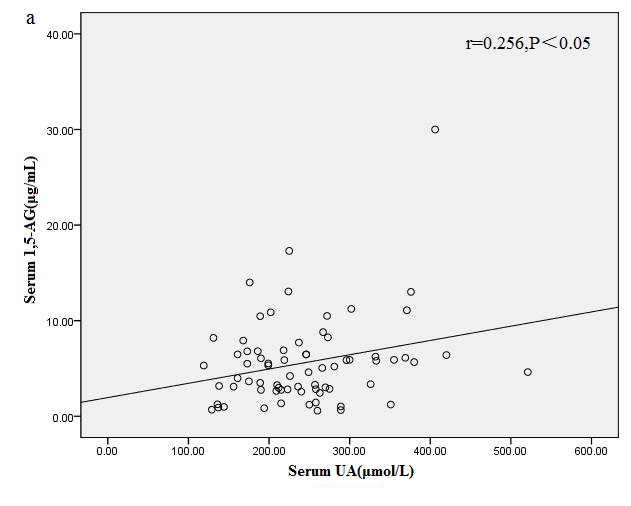


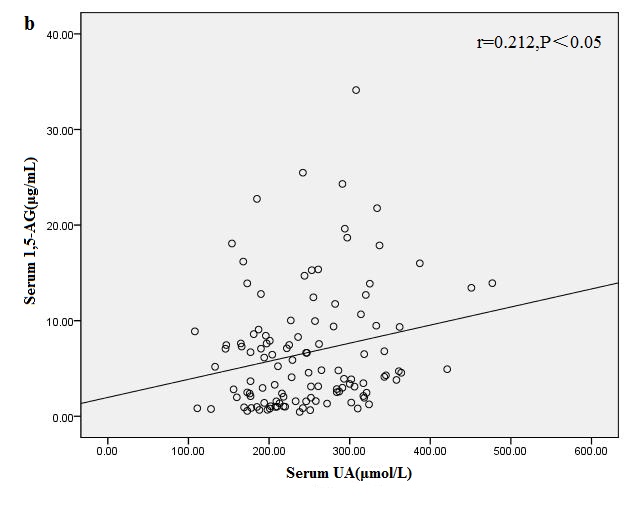

Supplement: Supplementary Materials — Supplementary Material Description: clinical characteristics of female participants based on menopausal status are shown in Table S1, and SUA levels among women with menopause were higher than those among premenopausal women. Table S2 shows the correlations between 1,5-anhydroglucitol and uric acid with different ACR. The association between SUA and 1,5-AG remains significant in diabetic patients with ACR < 300 mg/gCre (P < 0.01). The relationship between levels of serum 1,5-AG and serum UA in premenopausal women (r = 0.256,P < 0.05) and in postmenopausal women (r = 0.212,P < 0.05) are shown by Pearson's correlation coefficient in Figure S1. [file 4353075.f1.docx]
